# A mask-based peak-to-average power ratio reduction scheme for affine frequency division multiplexing systems using Volterra Series-Driven PolyNet

**DOI:** 10.1371/journal.pone.0354675

**Published:** 2026-07-28

**Authors:** Xiangxin Liu, Yushuai Zhang, Jianxin Guo, Rui Zhu, Feng Wang, Liping Wang

**Affiliations:** 1 School of Electronic Information, Xijing University, Xi’an, China; 2 Xi’an Key Laboratory of Intelligent Perception and Autonomous Navigation for Low-altitude Aircraft, Xi’an, China; Northwest Normal University, CHINA

## Abstract

Affine frequency division multiplexing (AFDM) demonstrates exceptional resilience to Doppler effects in doubly dispersive channels, making it a promising waveform for 6G high-mobility communications, but its high peak-to-average power ratio (PAPR) problem severely limits system energy efficiency. When migrated to AFDM, existing mask-based deep learning PAPR reduction schemes face the challenges of contextual loss caused by local sampling and the lack of physical interpretability in black-box models. Therefore, this paper proposes a physics-aware mask-based PAPR reduction scheme tailored for AFDM. First, a full-frame input strategy is introduced to exploit the global time-domain correlation of AFDM signals for precisely recovering the impaired symbols. Second, to address the nonlinear distortion induced by masking, a PolyNet-Volterra network is proposed by integrating Volterra series theory. Departing from the traditional design of blindly stacking layers, this model explicitly constructs first-order linear and third-order power feature layers, which substantially enhances the nonlinear reconstruction accuracy while avoiding overfitting. Simulation results demonstrate that, under a threshold of 0.9, the proposed scheme achieves a significant PAPR reduction of approximately 5.9 dB. Furthermore, requiring only about 165,000 parameters, the PolyNet-Volterra model comprehensively outperforms baseline models such as DNN and ResNet in terms of both bit error rate (BER) and mean squared error (MSE). Compared with the conventional PolyNet, the proposed model reduces the parameter and computational overhead, suggesting its potential as a low-complexity receiver-side reconstruction module for resource-constrained future wireless systems. Further hardware-oriented and over-the-air validation is still needed before drawing deployment-level conclusions.

## Introduction

With the rapid evolution of mobile communication technologies, the sixth-generation (6G) wireless network has become a global focal point for breakthrough technologies [1–3]. It aims to provide highly reliable and ubiquitous connectivity services for high-mobility scenarios, such as high-speed railways, low Earth orbit (LEO) satellite internet, and unmanned aerial vehicle (UAV) communications [4]. In such demanding scenarios, the high relative velocity between terminals and base stations induces significant Doppler shifts, causing the wireless channel to exhibit severe doubly selective fading characteristics. This leads to inter-symbol interference and frequency-selective fading, which makes channel estimation and equalization more difficult. To tackle these technical challenges, affine frequency division multiplexing (AFDM) has emerged as a novel multi-carrier modulation technique [5–7]. By incorporating the discrete affine Fourier transform (DAFT), AFDM can achieve full diversity gain in doubly dispersive channels. Furthermore, it not only delivers superior resilience to Doppler shifts compared to orthogonal frequency division multiplexing (OFDM), but also entails lower overhead for channel estimation and multi-user multiplexing than orthogonal time frequency space (OTFS) modulation [8–10].

AFDM is promising for high-mobility scenarios, but its time-domain waveform is still formed by the superposition of multiple chirp-modulated subcarriers. Therefore, the high peak-to-average power ratio (PAPR) problem still needs to be addressed [11,12]. When these signals with extremely high instantaneous peaks pass through a high power amplifier (HPA), they are driven into the saturation region, exceeding the linear amplification limit and suffering from clipping. This phenomenon inevitably causes in-band distortion, which elevates the bit error rate, and out-of-band radiation, which interferes with adjacent channels. Therefore, energy-constrained satellite, vehicular, and low-altitude communication scenarios provide a strong motivation for studying low-complexity AFDM PAPR reduction methods while maintaining communication reliability [13].

Currently, research on PAPR reduction is quite mature, encompassing traditional methods such as clipping, partial transmit sequence (PTS), and selective mapping (SLM) [14–16]. However, while clipping is simple and straightforward, it introduces irreversible nonlinear distortion, severely degrading the BER at the receiver [17,18]. Conversely, probabilistic schemes like PTS and SLM require the transmission of additional side information, and their computational complexity grows exponentially with the number of candidate sequences. In recent years, with the widespread application of deep learning in communication physical layer technologies [19], existing schemes [20–23] have successfully achieved PAPR reduction by leveraging deep neural networks (DNNs). Most notably, He et al. [24] proposed a mask-based PAPR reduction architecture for OFDM systems. This scheme masks a small fraction of high-peak symbols at the transmitter and uses a DNN at the receiver for signal reconstruction. In this way, PAPR reduction and signal recovery are handled in two separate steps, which improves the overall performance.

However, several critical issues in existing research remain unresolved. First, the input to their mask reconstruction neural network comprises only the symbols at the masked positions within the received signal, along with the indices of these positions. Specifically, rather than utilizing the entire received signal, the model’s input is strictly confined to the masked symbols. Although this input strategy reduces computational complexity, it discards much of the time-domain context around the masked samples. As a result, the network has limited information for estimating the local waveform trajectory, which may lead to similar reconstruction limits across different model architectures [25]. Furthermore, these existing black-box models ignore the physical mechanisms of communication signal distortion. They typically rely on blindly stacking massive numbers of neurons and network layers to approximate the nonlinear mapping, resulting in an enormous number of parameters and sluggish training convergence. This not only exacerbates the computational burden on the receiver but also fails to meet the stringent requirements of future 6G networks for low-latency and low-power communications [26–28].

To address the aforementioned dual challenges, this paper proposes a physics-aware mask-based PAPR reduction scheme tailored for AFDM systems. Initially, the scheme employs a dynamic masking mechanism to precisely locate high-peak regions within the signal, strategically replacing these high instantaneous power peaks to significantly reduce the PAPR at the source. Subsequently, a lightweight polynomial neural network designed based on Volterra series theory (termed PolyNet-Volterra) is utilized to perform nonlinear reconstruction of the impaired symbols within these peak regions. This innovative approach substantially lowers the PAPR while maintaining a low bit error rate (BER).

The main contributions of this paper are summarized as follows:

1. We introduce a mask-based PAPR reduction scheme into the AFDM system, validating its effectiveness under chirp modulation. We also extend the masked-symbol input strategy to a full-frame input strategy. This allows the network to use frame-level signal context when reconstructing the masked samples.
2. To handle the nonlinear amplitude distortion introduced by masking, we propose PolyNet-Volterra, a neural network model inspired by the Volterra series. The model uses power-related feature layers to improve interpretability. A mask-specific loss function is also used to improve reconstruction accuracy at high-energy masked samples.
3. Simulation results demonstrate that, under identical PAPR reduction levels, the proposed PolyNet-Volterra model achieves faster convergence and superior BER performance compared to conventional DNN and ResNet models, while drastically reducing the parameter count. These results indicate that PolyNet-Volterra is a promising low-complexity candidate for AFDM PAPR reduction, while its practical deployment still requires further validation under hardware and over-the-air conditions.

The remainder of this paper is organized as follows. The Related Work section reviews existing approaches. The AFDM System Model section introduces the system model. The Simulation Results Analysis section presents and analyzes the experimental results. Finally, the Conclusion section summarizes the study.

## Related work

In multi-carrier communication systems, the high peak-to-average power ratio (PAPR) has been a persistent technical challenge. For a continuous time-domain signal x(t),its PAPR is defined as the ratio of the peak power to the average power, which is mathematically expressed as:


$$\begin{array}{c} PAPR\{x(t)\}=\frac{\max_{0\leq t<T}|x(t){|}^{2}}{𝔼[|x(t){|}^{2}]} \end{array}$$ (1)

where $\max_{0\leq t<T}|x(t){|}^{2}$ denotes the maximum instantaneous power (i.e., the peak power) attainable by the signal within a continuous symbol period *T*, and $𝔼[|x(t){|}^{2}]$ represents the expected (average) power of the signal over the same period. An excessively high PAPR inevitably drives the signal into the nonlinear saturation region of the high-power amplifier (HPA), causing in-band signal distortion and out-of-band spectral regrowth. To address the severe PAPR issue in wireless multi-carrier systems, academia has proposed various solutions, including clipping, companding transforms, and precoding-based PAPR reduction schemes [29,30,31]. Existing research can generally be categorized into conventional signal processing methods and deep learning (DL)-based optimization approaches.

### Conventional PAPR reduction techniques

Conventional PAPR reduction techniques are quite mature and mainly include signal distortion techniques and probabilistic techniques.

Signal distortion techniques include methods such as clipping and companding. This approach directly compresses signal peaks through nonlinear operations, featuring simple implementation and high efficiency. However, such nonlinear processing destroys the orthogonality of the signal, introducing in-band distortion and out-of-band radiation, which leads to significant degradation of the bit error rate (BER) at the receiver. Although filtering can be applied to mitigate out-of-band radiation, it typically causes the peak regrowth phenomenon. In addition, techniques such as companding are also commonly used to smooth power peaks [32].

Probabilistic techniques include methods such as partial transmit sequence (PTS) and selective mapping (SLM). These methods generate multiple candidate signals by applying phase rotations to the signal in the frequency domain and select the one with the minimum PAPR for transmission. Although this category of techniques does not cause signal distortion, it requires executing multiple IFFT operations, and the computational complexity escalates exponentially with the number of candidate sequences. In AFDM systems, given the introduction of the discrete affine Fourier transform (DAFT) and additional chirp modulation parameters, the overhead of such multi-branch parallel computation would further increase, making it even more prohibitive for the system to tolerate.

### Existing deep learning-based PAPR reduction schemes

In recent years, deep learning has been widely applied to PAPR reduction tasks due to its powerful nonlinear fitting capabilities [33–35]. Some studies utilize autoencoders to completely replace traditional modulation and demodulation modules, jointly optimizing the PAPR and BER performance. For example, by designing a weighted loss function, the network is guided to generate low-PAPR waveforms. However, such tightly coupled architectures typically require collaborative training between the transmitter and the receiver, resulting in an enormous number of model parameters. Moreover, models trained for specific channels are difficult to generalize to the complex doubly selective fading scenarios of AFDM. Another category of methods employs deep learning to optimize the key parameters of traditional algorithms, such as learning the optimal phase factors in SLM or the extension vectors in ACE. Although these methods yield performance improvements, they remain confined by the high-complexity frameworks of traditional algorithms.

### Mask-based schemes and their limitations

In existing mask recovery schemes, the network typically takes only the impaired symbols at the masked positions as input. This design effectively reduces the input dimension of the model and performs excellently in OFDM systems. The underlying reason is that the subcarriers in OFDM exhibit relative independence, making local recovery sufficient to meet efficiency requirements. However, in AFDM systems, after the signal undergoes chirp modulation and the discrete affine Fourier transform (DAFT), a tighter coupling relationship emerges among the time-domain samples. This coupling originates from the non-orthogonality of the modulation scheme and the global nature of the transformation, causing the signal components to interweave with each other. If the recovery relies solely on the local information at the masked positions, it becomes difficult to fully exploit the rich contextual correlations embedded in the uncorrupted neighboring symbols, thereby degrading the accuracy and robustness of the recovery. This issue becomes particularly pronounced under high-noise or complex channel conditions.

Meanwhile, most existing reconstruction schemes employ generic fully connected neural networks (DNNs) for end-to-end recovery. Such generic architectures possess strong generalization and nonlinear fitting capabilities, enabling them to implicitly learn the nonlinear inverse transformations by deepening the network layers and adapt to various signal environments and impairment patterns. However, overly generic network structures are usually accompanied by massive parameter counts and high computational overhead. Such substantial parameter volumes and computational demands can lead to sluggish training processes and significantly increased memory footprints, posing feasibility challenges for deployment in real-time applications or on resource-constrained devices. In addition, generic DNNs may lack the effective encoding of prior knowledge in the signal processing domain and cannot be optimized for specific modulation or transformation characteristics. This limitation restricts the room for further improvements in model efficiency and performance. A comparison of existing schemes is presented in Table 1.

**Table 1:** Comparison of existing schemes.

Scheme Category	Representative Techniques	Transmitter Complexity	BER Degradation
Signal Distortion	Clipping,Companding	Very low	Severe
Probabilistic	PTS, SLM	Very high	None
Masking and DL Reconstruction	Scheme by He et al.	Very low	Slight

### AFDM system model

**Fig 1:**
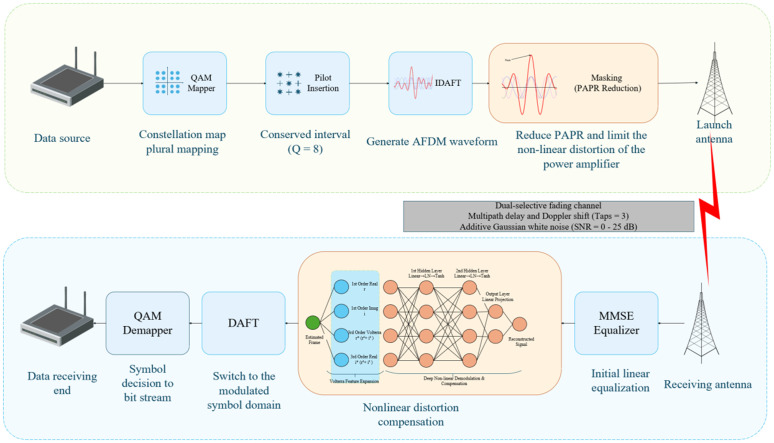
Deep learning-based AFDM system architecture.

This section details the proposed AFDM communication system architecture, which encompasses the complete signal transmission, reception, and processing link. At the transmitter, the bit stream sequentially undergoes quadrature amplitude modulation (QAM) mapping, pilot insertion, and inverse discrete affine Fourier transform (IDAFT) to generate the time-domain AFDM waveform. Subsequently, a time-domain masking module strategically intercepts and replaces the high instantaneous power symbols prior to transmission. This reduces the system PAPR at the source, effectively preventing the signal from being driven into the nonlinear saturation region of the power amplifier. The signal is then transmitted to the receiver over a doubly dispersive fading channel affected by multipath delays, Doppler shifts, and additive white Gaussian noise (AWGN). At the receiver, the signal captured by the antenna first undergoes preliminary linear channel equalization via a minimum mean square error (MMSE) equalizer before entering the PolyNet-Volterra neural network module cascaded thereafter. The PolyNet-Volterra module uses first-order and third-order power-related features inspired by the Volterra series to reconstruct the nonlinear distortion caused by masking before demodulation. Finally, the signal is transformed back to the symbol domain via the discrete affine Fourier transform (DAFT) and undergoes QAM demapping to recover the original data. The overall architecture of the proposed scheme is illustrated in Fig 1.

### Mask-based PAPR reduction mechanism

The core of the AFDM system lies in utilizing the discrete affine Fourier transform (DAFT) to process the signal, enabling it to maintain orthogonality in doubly dispersive channels. The time-domain transmitted signal of AFDM, denoted as $x=[x[0],x[1],\ldots ,x[N-1]{]}^{T}$, is generated by the inverse discrete affine Fourier transform (IDAFT):


$$\begin{array}{c} x=A^{-1}s=\Lambda_{c_{2}}F^{H}\Lambda_{c_{1}}s \end{array}$$ (2)

where $F^{H}$ denotes the standard inverse discrete Fourier transform (IDFT) matrix, and $s=[s_{0},s_{1},\ldots ,s_{N-1}{]}^{T}$ represents the transmitted symbol vector in the discrete affine Fourier transform (DAFT) domain. The matrices $\Lambda_{c_{1}}$ and $\Lambda_{c_{2}}$ are two diagonal chirp modulation matrices with their diagonal elements given by $e^{-j2\pi c_{1}n^{2}}$ and $e^{-j2\pi c_{2}n^{2}}$, respectively. The parameters *c*_1_ and *c*_2_ are selected based on the optimal parameter design criteria of AFDM as $c_{1}=\frac{2M+1}{2N}$ and $c_{2}=\frac{1}{2N}$, where *M* is an integer, to ensure that the signal achieves full diversity gain in the delay-Doppler domain. By expanding the aforementioned formula, the time-domain sample *x*[*n*] can be expressed as:


$$\begin{array}{c} x[n]=e^{-j2\pi c_{2}n^{2}}\sum_{k=0}^{N-1}\left(s[k]e^{-j2\pi c_{1}k^{2}}\right)e^{j\frac{2\pi }{N}nk} \end{array}$$ (3)

This equation reveals that, structurally, the signal is essentially an inverse discrete Fourier transform (IDFT) encapsulated by a dual-layer chirp term, namely the transmitter pre-processing $e^{-j2\pi c_{1}k^{2}}$ and the time-domain post-processing $e^{-j2\pi c_{2}n^{2}}$. From a physical perspective, this time-domain waveform is intrinsically a coherent superposition of N quadratically phase-modulated subcarriers at time index n. When the instantaneous phases of these subcarriers align in the complex plane at a specific moment due to constructive interference, their amplitudes accumulate linearly in phase. This causes the instantaneous power at that specific point to significantly exceed the average power of the entire frame. Although chirp modulation alters the phase distribution, it does not eliminate the fundamental mathematical core of multi-carrier linear summation. Consequently, AFDM inherits a high PAPR drawback similar to that of OFDM, where the peak power can theoretically reach N times the average power under conditions of extreme phase alignment.

A high PAPR not only degrades the operational efficiency of the high-power amplifier (HPA) but may also drive the signal into the nonlinear saturation region of the HPA, thereby inducing signal distortion. For AFDM signals employing chirp modulation, the nonlinear distortion from the HPA destroys the orthogonality of the chirps, resulting in severe inter-carrier interference (ICI) during DAFT demodulation at the receiver.

**Fig 2:**
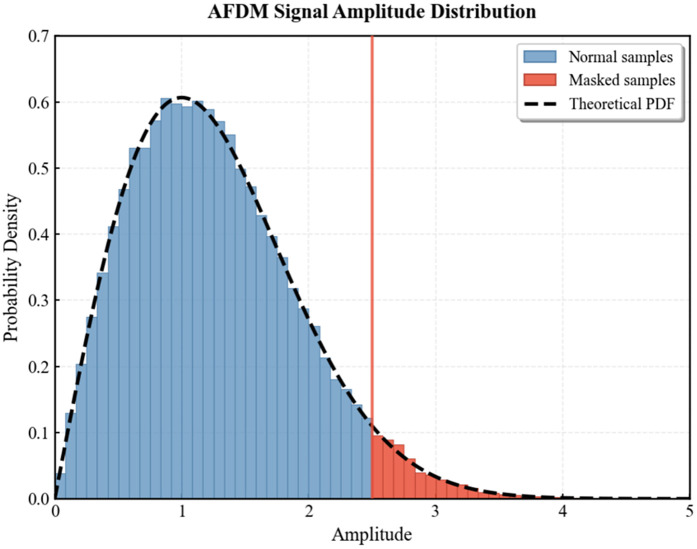
Probability density function of the AFDM signal amplitude.

**Fig 3:**
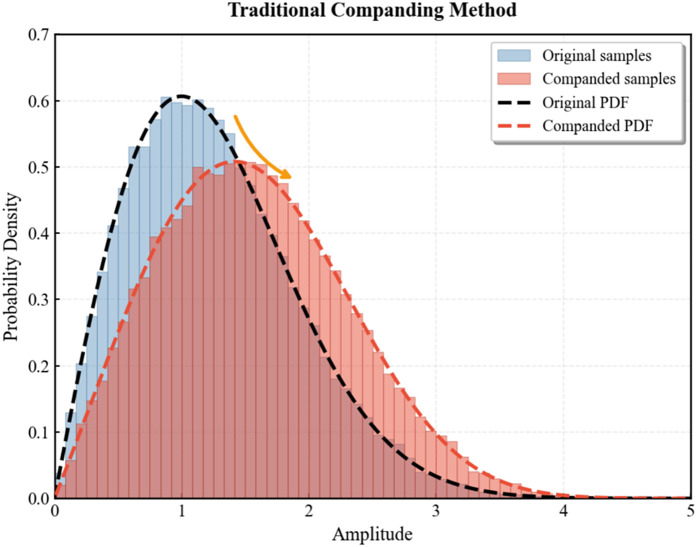
Impact of the conventional companding transform method on the signal.

To reduce the PAPR without incurring high computational complexity, this paper conducts a statistical analysis of the amplitude characteristics of AFDM signals. The probability density function of the AFDM signal amplitude exhibits a prominent long-tail distribution characteristic, as illustrated in Fig 2. A long-tail distribution is a probability distribution in statistics characterized by a higher frequency in its tail; that is, within a dataset, although the majority of data points are concentrated within a certain range, a significant number of rare events or outliers exist in the tail. This implies that only a minute fraction of symbols carry extremely high instantaneous amplitudes. This contrasts with conventional companding transform methods, which alter the entire signal, as shown in Fig 3. Based on this observation, this paper proposes employing a nonlinear processing mechanism based on time-domain masking. The core concept of this mechanism is to strategically replace the high-amplitude peaks in the time-domain signal directly at the transmitter, thereby limiting the peak power of the signal at the source. However, this nonlinear operation inevitably introduces in-band distortion, which is precisely the target that the deep learning network must recover in subsequent sections.

### Neural network-based signal recovery at the receiver

Before detailing the neural network architecture, this paper first formally defines the signal reconstruction task at the receiver from a mathematical perspective. Assuming the original time-domain AFDM signal is x, the masking operation at the transmitter can be essentially abstracted as a nonlinear function β(·). Then, the transmitted signal after mask processing, denoted as $x_{mask}$, can be expressed as:


$$\begin{array}{c} x_{mask}=\beta (x) \end{array}$$ (4)

After this signal propagates through the doubly selective fading channelHand is corrupted by additive white Gaussian noisez, the observed signalyat the receiver can be expressed as:


$$\begin{array}{c} y=H\beta (x)+z \end{array}$$ (5)

At the receiver, the signal first passes through a conventional channel equalizer (such as an MMSE equalizer) to eliminate the channel fading effects caused by multipath and Doppler shifts. After ideal equalization, the signal $\hat{x}$ can be approximately expressed as:


$$\begin{array}{c} \hat{x}\approx \beta (x)+\tilde{z} \end{array}$$ (6)

where $\tilde{z}$ is the residual noise after the equalization operation. Thus, it is evident that for a conventional receiver, even if the channel equalization is extremely perfect, the signal still retains the nonlinear distortion β(x) introduced by the masking operation. If symbol decision is performed directly at this stage, this nonlinear distortion will inevitably lead to a severe error floor.

Consequently, this paper designs a neural network ${ℱ}_{\theta }(\cdot )$ parameterized by θ to approximate the inverse function of the masking operation, $\beta^{-1}(\cdot )$, as closely as possible under complex noise interference. The core task of the network is to remove the effect of β from the impaired signal and reconstruct the original signal:


$$\begin{array}{c} x_{recover}={ℱ}_{\theta }(\hat{x})\approx \beta^{-1}\left(\beta (x)+\tilde{z}\right)\approx x \end{array}$$ (7)

In this receiver structure, neural reconstruction is performed after MMSE equalization. The MMSE equalizer first suppresses the dominant linear doubly selective channel effect, so that the neural network can focus on recovering the nonlinear distortion introduced by time-domain masking. This processing order makes the learning task more targeted and reduces the burden on the reconstruction network.

To approximate this inverse mapping with a compact model, we use the physical structure of the masking distortion to construct prior features for the network. Considering that the nonlinear amplitude distortion of the signal is strongly correlated with its instantaneous power, we design the following power-aware feature expansion vector and employ it as the physical feature input layer for the neural network ${ℱ}_{\theta }(\cdot )$. For any complex sample s=r+j·i within the received signal vector, where *r* is the real part, *i* is the imaginary part, and the instantaneous power is $P=r^{2}+i^{2}$, its feature expansion vector is defined as:


$$\begin{array}{c} \Phi (s)=[r,i,r\cdot P,i\cdot P] \end{array}$$ (8)

The retained feature terms are chosen from the dominant structure of amplitude dependent baseband distortion. The first order components *r* and *i* preserve the main linear signal content, while the third order power components *r*|*s*|^2^ and *i*|*s*|^2^ provide the lowest order nonlinear correction related to the instantaneous power. This treatment is consistent with Volterra inverse modeling and memory polynomial predistortion, where low order complex baseband terms such as *x* and *x*|*x*|^2^ are commonly used to model amplitude dependent RF distortion and inverse compensation. The second order terms are not included because they introduce even order cross products that are less consistent with the odd symmetric baseband distortion considered here, while also increasing the feature dimension.

The polynomial feature expansion is applied only inside the receiver-side reconstruction network after channel equalization; it does not modify the transmitter-side AFDM modulation matrix, chirp basis, or DAFT/IDAFT definition. Therefore, the expansion does not directly preserve or destroy AFDM chirp orthogonality. Its role is to reconstruct the masked time-domain samples before DAFT-domain demodulation.

Based on this physical prior, the complete forward propagation process of the neural network can be reformulated as a composite mapping of feature expansion and a multilayer perceptron (MLP):


$$\begin{array}{c} {ℱ}_{\theta }(\hat{x})={MLP}_{\theta }\left(\Phi (\hat{x})\right) \end{array}$$ (9)

Through this explicit construction, the network is no longer required to guess the patterns of nonlinear envelope distortion via deep neuron stacking. Instead, it performs weighted fusion directly based on the key physical features provided by Φ(·), which comprise the first-order fundamental components (*r*,*i*) containing the basic information of the original signal, and the third-order power distortion components (r·P,i·P). This architecture, combining feature engineering with a lightweight network, tremendously boosts the efficiency of approximating $\beta^{-1}(\cdot )$. In terms of mathematical formulation, this feature construction method draws inspiration from the nonlinear system modeling theory of the Volterra series [36]. For a memoryless nonlinear system, its output *y*(*n*) can be approximately expressed as an odd-order polynomial expansion of its input *x*(*n*):


$$\begin{array}{c} y(n)\approx \alpha_{1}x(n)+\alpha_{3}x(n)|x(n){|}^{2}+\alpha_{5}x(n)|x(n){|}^{4}+\cdots \end{array}$$ (10)

**Fig 4:**
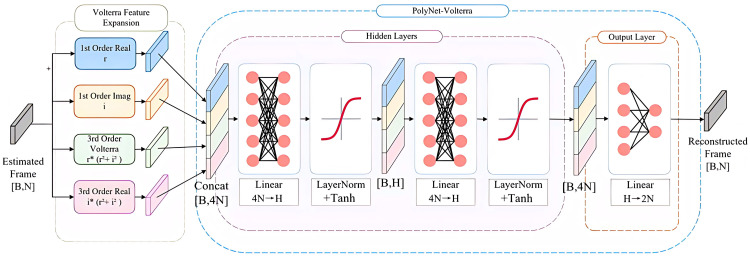
PolyNet-Volterra Network Architecture.

Based on the above theory, this paper designs the PolyNet-Volterra network, as shown in Fig 4. Unlike conventional DNNs, the proposed PolyNet-Volterra neural network adopts an architecture that combines physics-prior-based feature expansion with a deep multilayer perceptron (MLP). The network is designed to compensate for the nonlinear distortion induced by the masking operation. The entire forward propagation process is divided into three main stages:

1. The network receives an estimated complex signal frame of dimension *N* as input. Based on Volterra series theory, the network first decouples the signal and extracts four-dimensional physical features, including the first-order real part (*r*) and imaginary part (*i*), as well as the third-order nonlinear terms r·P and i·P constructed from the instantaneous power $P=r^{2}+i^{2}$. The four *N*-dimensional feature vectors are concatenated into a real-valued tensor of dimension 4*N*, which serves as the input to the subsequent network.

The full-frame input strategy is adopted because AFDM samples are globally coupled through chirp modulation and DAFT/IDAFT operations. Using only the masked positions discards neighboring and frame-level waveform context, which is important for reconstructing the trajectory around high-power peaks. The receiver therefore feeds the entire equalized AFDM frame into the reconstruction network and updates only the masked positions at the output.

For AFDM frames with sizes different from N = 64, the same mask-and-reconstruct principle can still be applied after adjusting the input/output dimension and retraining the network for the new frame size. The current fully connected implementation should therefore not be regarded as a size-free model, but as an architecture that can be resized and retrained for other AFDM frame lengths.

2. The concatenated high-dimensional features are fed into the MLP backbone network consisting of two fully connected layers. The first layer maps the 4N-dimensional input to a hidden space with H neurons. Each linear mapping is followed by a cascade of layer normalization and the Tanh activation function. This structure not only effectively mitigates the internal covariate shift problem, but also endows the network with the expressive capacity required to fit the complex nonlinear amplitude-phase distortion.

Tanh is used in the PolyNet variants because the polynomial expansion can create large feature magnitudes near masked peaks, and a bounded activation helps keep normalized complex-baseband reconstruction stable. GELU and SiLU/Swish are well-established smooth activation functions [37,38] and are reasonable alternatives, but they were not selected as the default here because their unbounded positive side may amplify large polynomial features unless additional normalization or tuning is added. A dedicated activation-function ablation, including GELU and SiLU, is a meaningful future extension.

The hidden dimension was selected as 256 because the AFDM simulations use N = 64 subcarriers and the PolyNet-Volterra expansion forms four real-valued features per subcarrier, giving an expanded feature dimension of 4N = 256. This width lets the first hidden layer preserve the full expanded physical-feature representation instead of imposing an immediate bottleneck, while keeping the model compact enough for fair comparison with the main baselines. The same 256-dimensional setting was then used for the principal neural baselines and PolyNet variants so that performance differences mainly reflect the feature design and architecture rather than a narrower proposed model.

3. The final linear layer compresses the hidden layer features to 2N dimensions. Finally, these 2N real numbers are reconstructed into an N-dimensional complex tensor, where the first half serves as the real part and the second half as the imaginary part, thereby outputting the restored AFDM signal frame with both low PAPR and low distortion characteristics.

## Simulation results analysis

### Simulation setup

To evaluate the performance of the proposed PolyNet-Volterra scheme in the AFDM system, a deep learning simulation platform based on PyTorch is built in this paper. The simulation architecture strictly follows the standard settings in the fundamental theoretical literature of the AFDM system [5–7]. The detailed simulation parameter configurations are listed in Table 2.

**Table 2:** Simulation parameter configuration.

Parameter	Value	Description
Total subcarriers (N)	64	Number of time/frequency-domain samples in a complete frame
Guard interval (Q)	8	Guard length for eliminating inter-symbol interference (ISI)
Data symbol length (D)	47	Number of symbols actually used for data transmission (calculated as N-2Q-1)
Modulation scheme	QPSK (M_mod = 4)	Basic data symbol mapping method
First chirp parameter	3/128	Calculated according to (2*1 + 1)/(2N)
Second chirp parameter	1/4096	Calculated according to 1.0/Nˆ2
Number of multipath components	3	Number of independent multipaths in the doubly selective Rayleigh fading channel
Maximum delay spread	2	Maximum multipath delay spread
Maximum Doppler shift	1	Number of subcarrier spacings corresponding to the maximum Doppler shift
Training SNR	U(5, 25) dB	Continuous SNR with uniform distribution generated dynamically online
Testing SNR	0, 5, 10, 15, 20, 25 dB	Six fixed discrete SNR testing points for performance evaluation
Global random seed	42	Fixed seed for PyTorch and NumPy to ensure reproducibility
Batch size	512	Number of parallel frames per forward propagation
Training epochs	150	Total training epochs for the neural network
Batches per epoch	50	Dynamically generates 50 batches per epoch via online generation
Optimizer	Adam	Adaptive Moment Estimation algorithm
Initial learning rate	0.001	Initial learning rate for the Adam optimizer
Learning rate scheduler	StepLR (step_size = 15, gamma = 0.5)	Halves the learning rate every 15 epochs
Loss function	Masked MSE	Calculates the mean squared error exclusively on target_frame[mask_idx]
Test set size	100,000 frames/SNR point	100 realizations * 1000 batch size

To ensure the optimal performance of the PolyNet-Volterra network in achieving nonlinear signal reconstruction within the AFDM system, this paper designs a comprehensive training framework encompassing a specific-region loss function, a dynamic SNR adaptation mechanism, and a numerical stability control strategy. Given the spatial sparsity of the masking operation, the traditional global mean square error (Global MSE) is prone to being dominated by minute noise errors at numerous unmasked positions, which consequently dilutes the network’s focus on recovering high-amplitude distortions. To address this, we propose a mask-specific region loss function, which calculates the reconstruction error exclusively at the masked symbol positions. This loss function is defined as follows:


$$\begin{array}{c} ℒ(\theta )=\frac{1}{\left|K\right|}\sum_{k\in K}{\left\|{\hat{s}}_{k}(\theta )-s_{k}\right\|}^{2} \end{array}$$ (11)

where *K* denotes the index set of the masked symbols, and |*K*| represents its cardinality. ${\hat{s}}_{k}$ and $s_{k}$ denote the reconstructed output of the network and the original transmitted symbol at position *k*, respectively, while θ represents the set of network parameters. Through this focusing mechanism, the backpropagated gradients can bypass the limitations of the activation function’s gradients–much like the cross-entropy loss in network training–and guide the network to optimize its nonlinear reconstruction capability based directly on the discrepancy between the predicted and actual values. To endow the model with robust generalization capabilities under time-varying channel conditions, we abandon the traditional paradigm of fixed-SNR training in favor of a dynamic SNR adaptation mechanism. During each training batch, the signal-to-noise ratio (SNR) is randomly sampled from a uniform distribution $U({SNR}_{\min},{SNR}_{\max})$, where ${SNR}_{\min}=5\;dB$ and ${SNR}_{\max}=25\;dB$. This data augmentation strategy forces the network to learn the intrinsic structural features of the signal rather than memorizing specific noise patterns, thereby significantly enhancing the model’s adaptability across diverse channel environments.

All numerical results are obtained from synthetic AFDM simulations generated from the system model described above. The global random seed is fixed to 42 for the main experiments. The implementation uses Python, PyTorch, NumPy, and Matplotlib. No early stopping is used; all enabled neural models are trained for the scheduled 150 epochs with Adam optimization and the StepLR learning-rate scheduler. The fixed training workload, random seeds, model configurations, and numerical data behind the added tables are provided to improve reproducibility.

### Training convergence results

**Fig 5:**
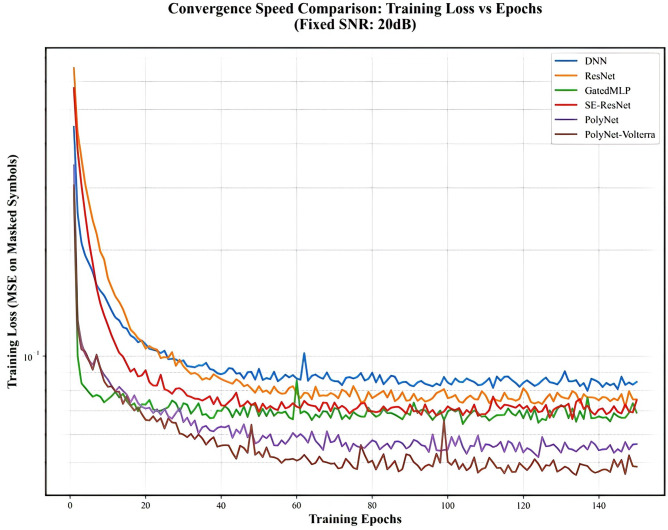
Convergence process during training under a fixed SNR (20 dB) environment.

The convergence process under a fixed signal-to-noise ratio (SNR) environment of 20 dB is illustrated in Fig 5. Since the background noise power remains constant across all training epochs, the random interference induced by noise is eliminated, resulting in curves that exhibit a clear and smooth downward trend. Consequently, the fitting limits and convergence speeds of the various models are distinctly observable. It is evident that the traditional DNN and ResNet converge slowly and suffer from higher error lower bounds, whereas the proposed PolyNet-Volterra network, after rapidly plateauing, consistently maintains the lowest error level among all evaluated models. This demonstrates that incorporating the Volterra physical mechanism effectively enhances both the learning efficiency and reconstruction accuracy of the network.

**Fig 6:**
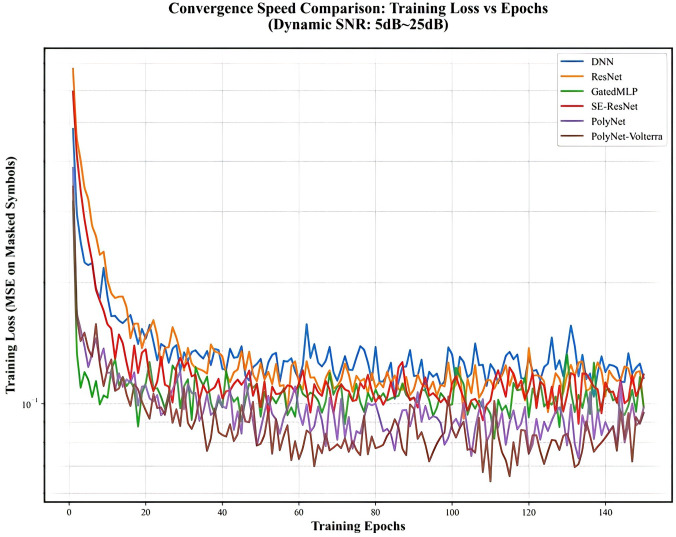
Convergence process during training under a dynamic SNR (5 dB to 25 dB) environment.

The training state under a dynamic SNR environment, which randomly fluctuates between 5 dB and 25 dB, is depicted in Fig 6. Due to the severe fluctuations in the background noise intensity of the input data for each batch, the mean square error (MSE) calculated by the network also exhibits substantial variations, manifesting as dense sawtooth oscillations. Although the curves fluctuate strongly, PolyNet-Volterra remains lower than the other tested models over most training epochs. This result suggests that the proposed model can maintain stable reconstruction performance under varying SNR conditions.

Empirical verification confirms that this dynamic SNR training method does not significantly increase the computational burden. The total number of training samples remains constant; the samples are simply generated online to be uniformly distributed across various SNRs and channel conditions. Since the number of training samples is unchanged and the samples are generated online, dynamic SNR training does not add an obvious training-time burden. It improves generalization without increasing the dataset size.

### Model selection and evaluation

To determine the optimal signal reconstruction architecture, this paper is not limited to a single model. Instead, we conduct extensive reproduction and comparative experiments (training from scratch) on various representative network architectures in the current deep learning domain, as shown in Table 3:

**Table 3:** Benchmark model configurations and comparison rationale.

Model	Input features	Core architecture	Benchmark role	Main comparison purpose	Norm & act
DNN	128	MLP	Generic black-box baseline	Non-prior fully connected reconstruction	BatchNorm + ReLU
ResNet	128	ResBlock	Residual-learning baseline	Effect of skip connections	BatchNorm + ReLU
GatedMLP	128	Gating	Gated-feature baseline	Effect of multiplicative feature selection	BatchNorm + Sigmoid
SE-ResNet	128	SE-Res	Feature-recalibration baseline	Effect of channel-wise feature weighting	BatchNorm + ReLU
PolyNet	448	Shallow MLP	Blind polynomial baseline	Broad polynomial expansion without selected Volterra subset	LayerNorm + Tanh
PolyNet-Volterra	256	Shallow MLP	Proposed physics-aware model	Compact selected first/third-order features	LayerNorm + Tanh

The benchmark models were selected to represent complementary lightweight reconstruction families under a common fully connected input-output interface. The DNN baseline reflects a generic black-box multilayer perceptron; ResNet tests residual learning; GatedMLP examines gated feature modulation; SE-ResNet adds channel-wise recalibration; and PolyNet provides a blind polynomial-expansion comparison. This set allows the effect of the physically selected Volterra-inspired first-plus-third-order features to be isolated under the same data, optimizer, batch size, epoch number, and evaluation metrics. More recent attention-based, sequence, graph, or generative reconstruction models are relevant future baselines, but they introduce different inductive biases and usually larger hardware-dependent costs that should be evaluated in a separate benchmarking study.

The computational complexity and performance scores of the various models are also quantified, with the experimental results presented in Table 4. This demonstrates that the proposed PolyNet-Volterra network possesses an overwhelming advantage in parameter efficiency. Due to its fully connected nature, the traditional DNN typically involves a massive number of parameters, leading to high computational load and memory consumption during training. In contrast to ResNet or DNN, the PolyNet-Volterra network requires significantly fewer parameters to achieve superior MSE and BER, obtaining the highest comprehensive performance score among all tested models. This indicates that the proposed scheme provides a favorable accuracy-complexity tradeoff among the tested simulation baselines. Its practical deployment performance should still be evaluated with hardware platforms and measured RF signals.

**Table 4:** Computational complexity and performance scores of different models ($Score=10^{6}/(BER\times Params\times MSE)$).

ModelName	Params	BER@20dB	MSE	Score
DNN	330880	0.00312	0.330896	2792.4
ResNet	463744	0.00264	0.315215	2591.3
GatedMLP	363648	0.00213	0.295652	4366.8
SE-ResNet	512896	0.00233	0.305568	2738.5
PolyNet	214656	0.00176	0.271149	9761.9
PolyNet-Volterra	165504	0.00155	0.255198	15275.0

BatchNorm is used in the generic DNN, ResNet, GatedMLP, and SE-ResNet baselines because these models follow common batch-statistics-based MLP/residual designs, where mini-batch normalization helps stabilize deep real-valued hidden layers [39]. LayerNorm is used in the PolyNet variants because the polynomial feature distribution is frame dependent and can vary strongly with the masked peaks; normalizing within each sample is therefore more suitable for the frame-level polynomial representation [40]. All enabled models use the same optimizer, batch size, epoch number, SNR setting, and evaluation protocol.

### BER performance analysis

**Fig 7:**
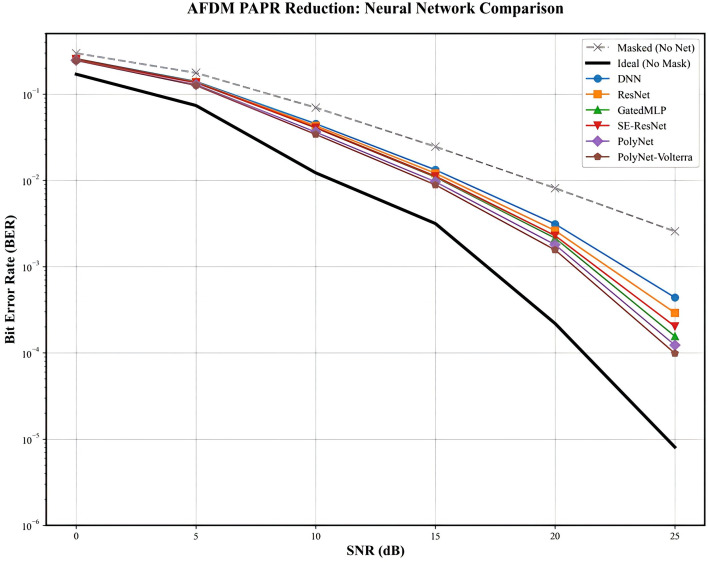
Bit Error Rate (BER) performance comparison under different SNRs (0-25 dB).

The error rates and performance comparison of the various schemes tested in a random SNR environment ranging from 0 to 25 dB are illustrated in Fig 7 and Table 5. Among all comparative models, the BER curve of the PolyNet-Volterra network is the closest to the ideal curve. Particularly in the high-SNR region (e.g., 20 dB), compared with traditional ResNet and DNN models, its BER is further reduced while the number of parameters drops substantially. This improvement is attributed to its unique physics-aware feature layer, which can accurately reversely simulate and eliminate the nonlinear distortion introduced by the masking operation.

**Table 5:** BER of each scheme under different SNRs (0-25 dB).

ModelName	BER@5dB	BER@10dB	BER@15dB	BER@20dB	BER@25dB
DNN	140.689e-03	45.447e-03	13.275e-03	3.123e-03	0.440e-03
ResNet	138.346e-03	43.019e-03	12.113e-03	2.620e-03	0.292e-03
GatedMLP	137.289e-03	40.677e-03	10.934e-03	2.143e-03	0.156e-03
SE-ResNet	135.783e-03	41.048e-03	11.209e-03	2.289e-03	0.203e-03
PolyNet	129.078e-03	36.409e-03	9.656e-03	1.799e-03	0.123e-03
PolyNet-Volterra	126.473e-03	34.203e-03	8.891e-03	1.564e-03	0.099e-03

### Volterra order ablation study and minimalist architecture justification

In the previous comparisons, the PolyNet-Volterra network demonstrated outstanding signal recovery capabilities with an extremely low number of parameters. This section delves into its underlying mechanisms to verify the scientific validity of relying exclusively on the 1st-order (*r*,*i*) and 3rd-order (r·P,i·P) physics-prior features. This paper comprehensively analyzes the effectiveness of this minimalist architecture from multiple perspectives, including multi-dimensional feature ablation, feature-distortion correlation, power-dependent error distribution, and neural network interpretability.

### Ablation of physics-prior features and parameter efficiency analysis

To explicitly determine the independent contribution of the features at each order, we construct various feature-subset models to compare their mean square errors and parameter efficiencies. The constructed feature-subset models are detailed in Table 6:

**Table 6:** Feature-subset models.

Model Name	Feature Description
DNN Baseline	Only inputs the concatenation of real and imaginary parts.
Standard PolyNet (Blind 1–3 Order)	Blindly expands 9 sets of fully cross-coupled polynomial features from 1st to 3rd order
Linear Only	Contains only 1st-order linear features
3rd-Order Only	Contains only 3rd-order features
Power Aug.	Concatenates 1st-order features with the power term
PolyNet-Volterra (Ours)	Our proposed feature subset extracted based on physical priors

**Fig 8:**
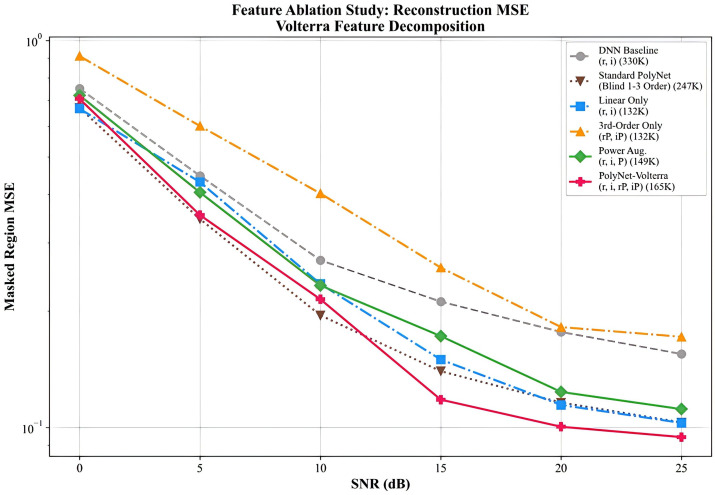
Comparison of reconstruction MSE in the masked region across different feature combinations.

Relying exclusively on the third-order features results in the highest MSE, indicating that the first-order linear features serve as the cornerstone of signal reconstruction. Despite possessing a massive number of parameters, the DNNBaseline lacks physical priors, rendering its performance even inferior to that of the simplest LinearOnly model. Simply introducing an independent power term (PowerAug) fails to effectively enhance performance, which demonstrates that the network struggles to autonomously couple phase and power. Furthermore, although the StandardPolyNet, which incorporates all first- to third-order cross-terms, exhibits improvements in MSE, its performance in the high-SNR region remains constrained by feature redundancy, accompanied by a substantially larger parameter count. These results support the compact first and third order design used in PolyNet-Volterra. The broader first-to-third-order feature set increases the model size, but it does not provide a better high-SNR accuracy and complexity balance than the selected Volterra subset. The specific comparisons are illustrated in Fig 8.

**Fig 9:**
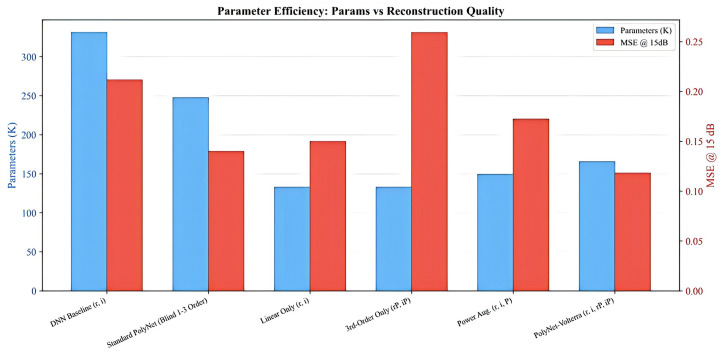
Histogram of parameter efficiency at 15 dB.

In contrast, the proposed PolyNet-Volterra exclusively retains four groups of core physical features (r,i,r·P,i·P), as depicted in Fig 9. While drastically compressing the parameter count to 165K under a 15 dB environment, it simultaneously achieves the lowest MSE across all SNR levels. This succinctly and powerfully verifies the correctness of utilizing Volterra physical priors to eliminate redundant polynomials, thereby constructing a minimalist yet highly efficient compensation architecture.

### Correlation analysis between features and nonlinear distortion

**Fig 10:**
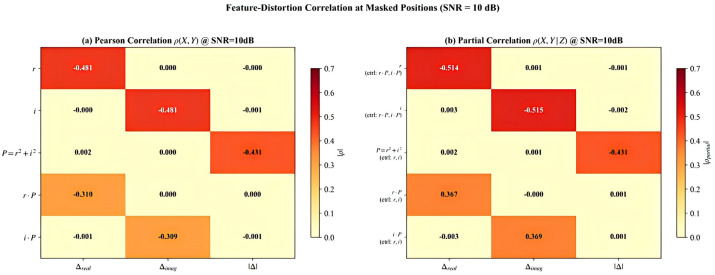
Heat map analysis of the correlation between various input features and nonlinear distortion errors at a 10 dB signal-to-noise ratio.

**Fig 11:**
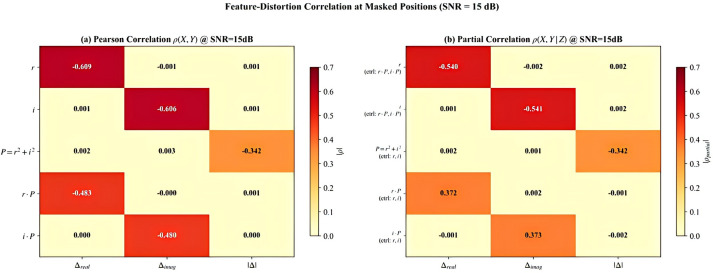
Heat map analysis of the correlation between various input features and nonlinear distortion errors at a 15 dB signal-to-noise ratio.

**Fig 12:**
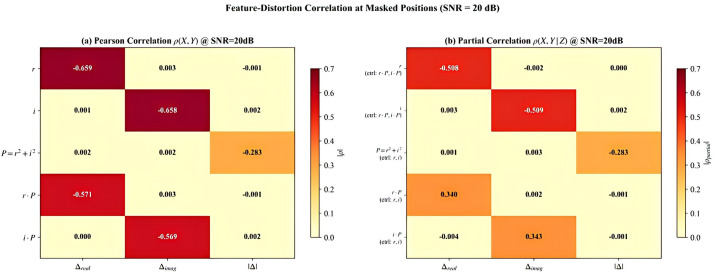
Heat map analysis of the correlation between various input features and nonlinear distortion errors at a 20 dB signal-to-noise ratio.

We calculate the Pearson correlation coefficients and partial correlation coefficients between the model input features and the actual distortion errors induced by the masking operation. As the SNR increases from 10 dB to 20 dB, the Pearson correlation between the features and the distortion errors gradually strengthens, as illustrated in Fig 10, Fig 11, and Fig 12. According to the evaluation criteria for Pearson correlation strength, an absolute value between 0.4 and 0.6 indicates a moderate correlation. Notably, the absolute value of the correlation coefficient between the third-order features and the errors reaches 0.571 at 20 dB, falling precisely into the moderate correlation range and exhibiting a considerably significant correlation. The partial correlation analysis on the right further confirms that even after controlling for the influence of the first-order features, the third-order features still maintain an independent and significant correlation. This intuitively explains the necessity of introducing the third-order physical features to accurately compensate for nonlinear distortion.

### Physical validation of power-dependent reconstruction errors

**Fig 13:**
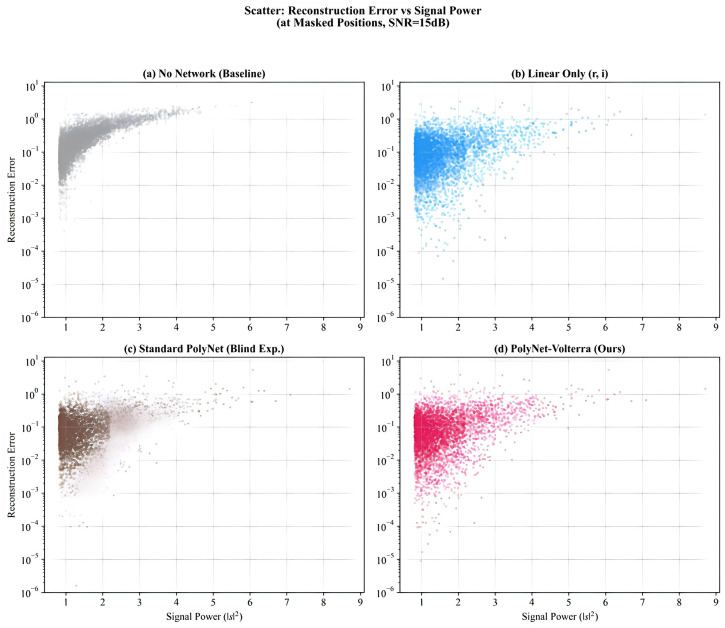
Scatter plot of the reconstruction error in the masked region versus the original signal power (SNR = 15 dB).

The essence of clipping or amplifier distortion lies in the fact that the degree of signal distortion is highly dependent on instantaneous power; namely, high-power peaks suffer severe impairment, whereas low-power signals essentially maintain linearity. We extract the scatter relationship between the prediction error and the original signal power |*s*|^2^ at an SNR of 15 dB, as shown in Fig 13. In Fig 13(a), the original error escalates exponentially with the increase in signal power. Fig 13(b) reveals that although the LinearOnly model lowers the overall error baseline, its error reduction in the left region of the coordinate axis is negligible. In Fig 13(c), constrained by low learning efficiency due to an excess of non-independent features, the StandardPolyNet still exhibits a pronounced divergent scatter distribution across the entire power range. In contrast, Fig 13(d) demonstrates that the PolyNet-Volterra outperforms the other models, validating that its nonlinear terms (r·P,i·P) can better manage the dynamic error envelope induced by clipping peak suppression and nonlinear saturation.

### Data-driven perspective: gradient response and weight allocation

**Fig 14:**
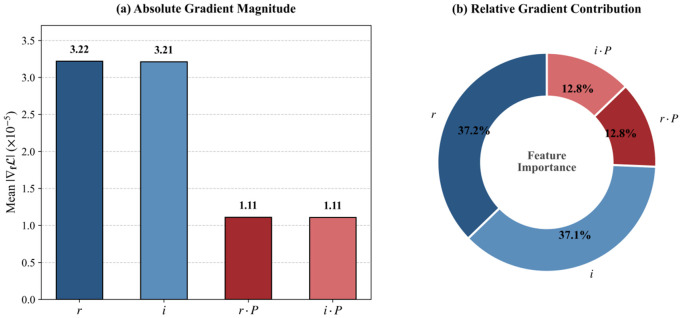
Gradient magnitude and relative contribution of the input features.

To verify the self-adaptive mechanism of this minimalist architecture in a data-driven environment, this paper unveils the black box of the neural network and analyzes the gradient importance of the features along with the first-layer weight distribution. The results are illustrated in Fig 14.

**Fig 15:**
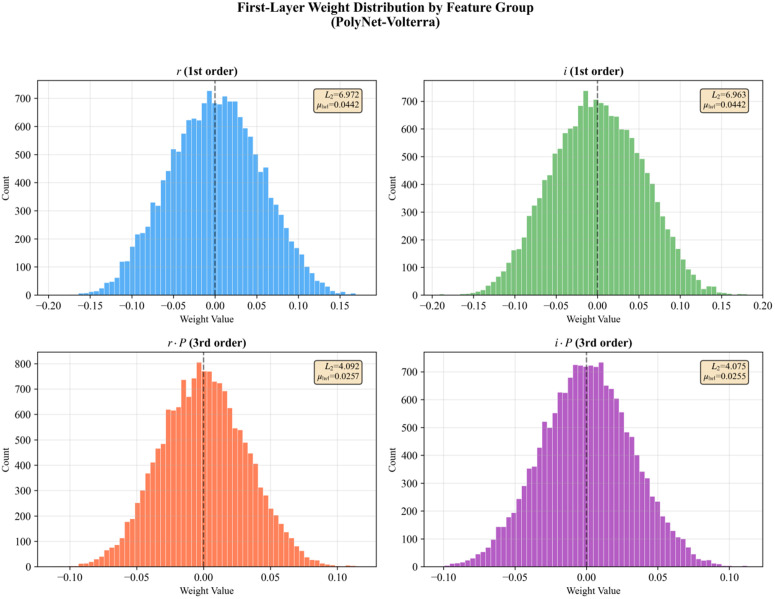
Feature weight distribution of the first-layer hidden neurons.

During the backpropagation process, the network not only allocates the primary gradient flow–accounting for approximately 74.3%–to the first-order linear features to ensure the fundamental signal reconstruction performance, but it also reserves a stable and significant optimization gradient–totaling 25.6%–for the third-order features. The weight distribution histogram in Fig 15 further indicates that upon convergence, the weights of the third-order features exhibit a healthy Gaussian-like distribution. Their *L*_2_ norm remains above 4.0, showing absolutely no signs of redundant failure such as collapsing toward zero under regularization. From the perspective of low-level network optimization, this thoroughly validates the effectiveness and indispensability of every single physical feature within the minimalist architecture.

Fig 15 shows that the first-layer weights are distributed over both the first-order features and the third-order power-coupled features. The linear real and imaginary components carry the largest weight energy, while the nonlinear rP and iP terms also keep clear nonzero contributions. This result suggests that the network does not simply rely on a black-box mapping, but uses the manually constructed low-order polynomial features during masked-sample reconstruction. Since these features are close in form to the basis terms used in truncated Volterra-type nonlinear inverse modeling [41,42], the proposed architecture can be viewed as a lightweight Volterra-inspired learned inverse approximation. It should not be regarded as a fixed polynomial kernel regression model, because the expanded features are still processed by learned nonlinear layers and optimized end to end for the AFDM reconstruction task.

### Reconstruction error analysis in the masked region

**Fig 16:**
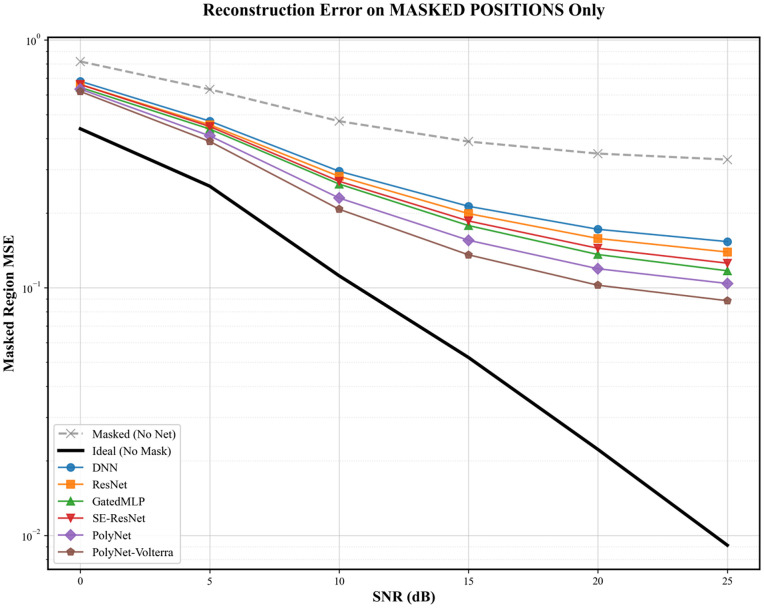
Mean square error of various schemes at the masked positions.

To delve deeper into the specific performance of different network models in signal recovery, the mean square error (MSE) comparison of various schemes at the masked positions is illustrated in Fig. 16.

Unlike the traditional global MSE metric, this paper calculates the reconstruction error exclusively for the signal samples that were replaced by the mask at the transmitter. This metric effectively eliminates the interference from unmasked normal signal samples, precisely measuring the accuracy of the neural network in recovering the lost peak information.

A pronounced performance stratification phenomenon can be observed from the figure, where the gray dashed line represents the error of the received signal without any processing. Since the peaks are replaced by non-peak symbols, an inherent amplitude and phase deviation exists at these positions. Consequently, its MSE consistently remains at a high level and exhibits negligible variation with the SNR, indicating that the primary source of error is the nonlinear distortion at the transmitter rather than channel noise. Compared to the baseline, traditional deep learning models significantly reduce the MSE, demonstrating their capacity for signal repair to some extent. However, in the high-SNR region, their MSE curves tend to plateau, suggesting that these models struggle to further eliminate residual nonlinear errors. The MSE curve of the proposed PolyNet-Volterra model ultimately reaches the lowest error level. This implies that the signal peaks predicted by this model are the closest to the originally transmitted signals.

This MSE result is highly consistent with the aforementioned BER performance, explaining the source of the PolyNet-Volterra’s advantage from a signal processing perspective: by utilizing the Volterra series features to more accurately fit the nonlinear amplitude compression process, the model achieves higher-fidelity waveform reconstruction at the physical layer, which subsequently translates into a lower bit error rate at the demodulation end.

### Additional validation and practical notes

**Fig 17:**
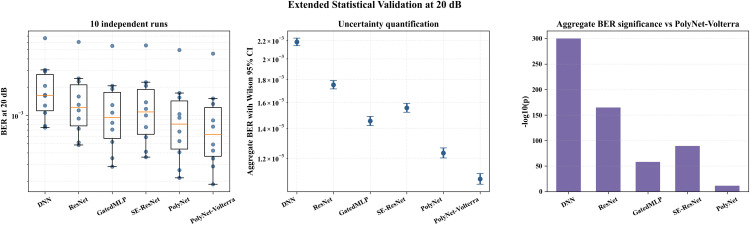
Extended statistical validation at 20 dB, including repeated-run BER distributions, Wilson confidence intervals, and aggregate significance levels.

To enhance the quantitative comparison, this paper conducted multiple independent statistical analyses. Table 7 reports 10 independent Monte Carlo simulation runs, each using 10,000 AFDM frames and an independent random seed, so the repeated-run variability can be seen directly. Table 8 reports aggregate uncertainty quantification using Wilson 95% confidence intervals for BER and run-level 95% confidence intervals for masked-region MSE. Table 9 reports formal significance testing against PolyNet-Volterra using an aggregate two-proportion z test for BER and a paired sign test across independent runs. Fig. 17 summarizes the repeated-run distribution, uncertainty intervals, and significance levels.

**Table 7:** Repeated independent Monte Carlo variability at 20 dB.

Model	Independent runs	BER mean ± SD	Masked MSE mean ± SD
DNN	10 x 10000 frames	2.182e-03 ± 1.760e-03	0.1657 ± 0.0088
ResNet	10 x 10000 frames	1.750e-03 ± 1.659e-03	0.1495 ± 0.0090
GatedMLP	10 x 10000 frames	1.453e-03 ± 1.544e-03	0.1335 ± 0.0092
SE-ResNet	10 x 10000 frames	1.554e-03 ± 1.548e-03	0.1405 ± 0.0091
PolyNet	10 x 10000 frames	1.232e-03 ± 1.409e-03	0.1102 ± 0.0080
PolyNet-Volterra	10 x 10000 frames	1.078e-03 ± 1.300e-03	0.0974 ± 0.0072

**Table 8:** Uncertainty quantification with BER and masked-region MSE confidence intervals.

Model	Aggregate BER with Wilson 95% CI	Wilson half-width	Masked MSE with 95% CI
DNN	2.182e-03 [2.140e-03, 2.225e-03]	4.219e-05	0.1657 [0.1594, 0.1721]
ResNet	1.750e-03 [1.713e-03, 1.788e-03]	3.779e-05	0.1495 [0.1431, 0.1559]
GatedMLP	1.453e-03 [1.419e-03, 1.488e-03]	3.444e-05	0.1335 [0.1269, 0.1400]
SE-ResNet	1.554e-03 [1.519e-03, 1.590e-03]	3.562e-05	0.1405 [0.1340, 0.1470]
PolyNet	1.232e-03 [1.201e-03, 1.264e-03]	3.172e-05	0.1102 [0.1045, 0.1159]
PolyNet-Volterra	1.078e-03 [1.049e-03, 1.108e-03]	2.967e-05	0.0974 [0.0922, 0.1026]

**Table 9:** Formal significance tests against PolyNet-Volterra at 20 dB.

Comparison	Mean BER difference	Aggregate BER test p	Paired sign test p
DNN vs PolyNet-Volterra	1.104e-03	<1.0e-300	1.953e-03
ResNet vs PolyNet-Volterra	6.719e-04	2.030e-165	1.953e-03
GatedMLP vs PolyNet-Volterra	3.751e-04	7.735e-59	1.953e-03
SE-ResNet vs PolyNet-Volterra	4.764e-04	3.163e-90	1.953e-03
PolyNet vs PolyNet-Volterra	1.538e-04	3.844e-12	2.148e-02

**Fig 18:**
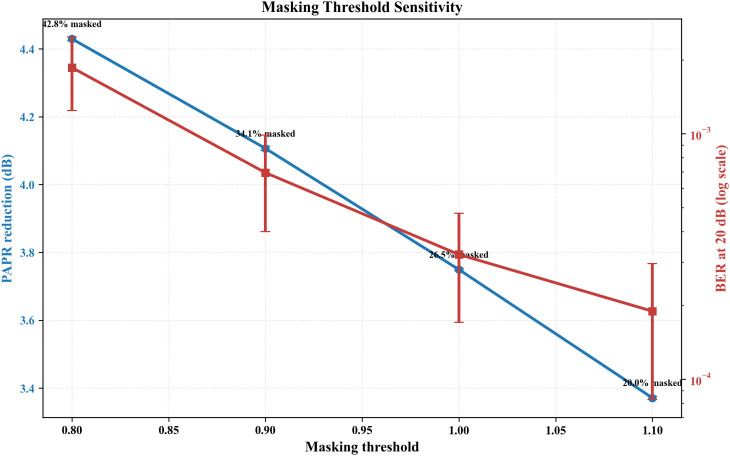
Sensitivity of PAPR reduction and reconstruction performance to the masking threshold at 20 dB.

The masking threshold is a sensitivity parameter that controls how aggressively the transmitter compresses large time-domain peaks. Table 10 and Fig 18 give the sensitivity trend. When the threshold is reduced, peak suppression becomes stronger, but the reconstruction problem becomes harder. When the threshold is increased, the reconstruction burden is relaxed, but the peak-compression effect becomes weaker.

**Table 10:** Sensitivity to the masking threshold at 20 dB.

Threshold	PAPR reduction (dB)	BER mean ± SD	Masked MSE mean
0.8	4.43	1.860e-03 ± 6.180e-04	0.1135
0.9	4.11	6.940e-04 ± 2.944e-04	0.0992
1.0	3.75	3.221e-04 ± 1.515e-04	0.0930
1.1	3.37	1.894e-04 ± 1.065e-04	0.0966

**Fig 19:**
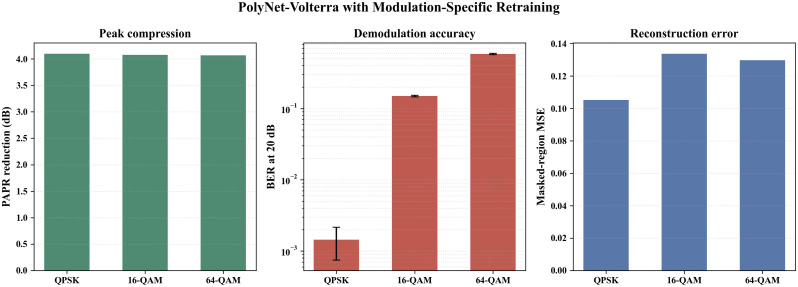
High-order modulation performance after modulation-specific PolyNet-Volterra training.

To examine high-order modulation more directly, PolyNet-Volterra was trained and evaluated under QPSK, 16-QAM, and 64-QAM, respectively. Table 11 and Fig 19 shows the demodulation accuracy and masked-region reconstruction error for different modulation schemes after modulation-specific training. As the constellation becomes denser, the decision regions become narrower, leading to a higher bit error rate (BER). Meanwhile, the masked-region mean square error (MSE) also increases. Since channel coding, interleaving, and BER-oriented detector optimization are not included in this study, the BER and MSE values remain relatively high under dense constellations. Even so, the results suggest that high-order modulation can still be supported through retraining.

**Table 11:** High-order modulation experiment with modulation-specific PolyNet-Volterra training.

Modulation	Training setting	PAPR reduction (dB)	BER mean ± SD	Masked MSE mean
QPSK	separate training	4.10	1.450e-03 ± 7.047e-04	0.1052
16-QAM	separate training	4.08	1.497e-01 ± 3.476e-03	0.1338
64-QAM	separate training	4.07	5.833e-01 ± 1.360e-02	0.1297

**Fig 20:**
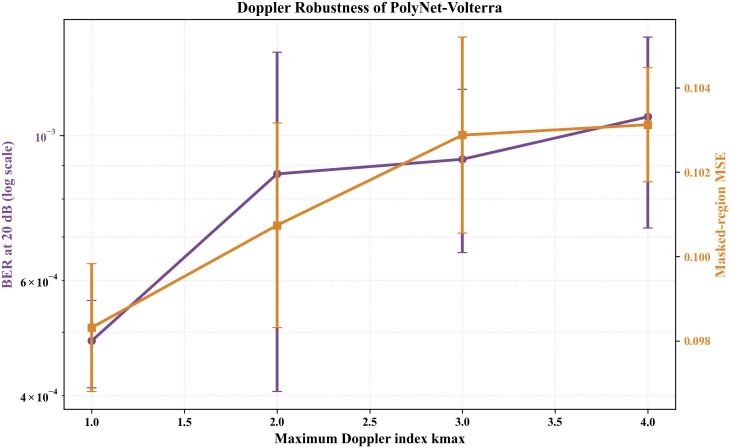
Doppler robustness of PolyNet-Volterra under increased maximum Doppler index.

The Doppler robustness test in Table 12 and Fig 20 increases the maximum integer Doppler index from 1 to 4 while keeping the same receiver structure. The BER and MSE increase moderately, indicating that the full-frame reconstruction remains usable under the tested higher-Doppler simulated channels. Regarding unseen channel models, this Doppler stress test provides an initial robustness check, but alternative channel families, model mismatch, and measured channels should be evaluated before making broad channel-generalization claims.

**Table 12:** Doppler robustness of PolyNet-Volterra at 20 dB.

Maximum Doppler index kmax	BER mean ± SD	Masked MSE mean
1	4.851e-04 ± 7.388e-05	0.0983
2	8.734e-04 ± 4.676e-04	0.1007
3	9.196e-04 ± 2.572e-04	0.1029
4	1.068e-03 ± 3.462e-04	0.1031

Table 13 reports parameter counts, linear-layer FLOPs per AFDM frame, and RTX 3090 GPU inference latency. The latency benchmark measures only the neural reconstruction module and excludes channel equalization and data generation. Single-frame latency and batch-256 latency are reported separately because they correspond to different inference modes.

**Table 13:** Complexity and RTX 3090 GPU inference latency of neural reconstruction models.

Model	Params	Linear FLOPs/frame	Single-frame GPU latency (ms)	Batch-256 GPU latency/frame (ms)
DNN	330880	0.655 M	0.3337	0.0014
ResNet	463744	0.918 M	0.6771	0.0031
GatedMLP	363648	0.721 M	0.4513	0.0019
SE-ResNet	512896	1.016 M	0.9229	0.0040
PolyNet	214656	0.426 M	0.2686	0.0010
PolyNet-Volterra	165504	0.328 M	0.2626	0.0011

Training wall-clock time was organized as a separate hardware-dependent metric. Table 14 reports the measured 150-epoch training time on the NVIDIA GeForce RTX 3090 GPU used for the main training runs.

**Table 14:** Measured RTX 3090 GPU training time for the 150-epoch training schedule.

Model	GPU platform	Training schedule	Wall-clock training time
DNN	NVIDIA GeForce RTX 3090	150 epochs, 50 batches/epoch, batch size 512	47.61 s (0.7935 min)
ResNet	NVIDIA GeForce RTX 3090	150 epochs, 50 batches/epoch, batch size 512	65.87 s (1.0978 min)
GatedMLP	NVIDIA GeForce RTX 3090	150 epochs, 50 batches/epoch, batch size 512	55.10 s (0.9184 min)
SE-ResNet	NVIDIA GeForce RTX 3090	150 epochs, 50 batches/epoch, batch size 512	76.68 s (1.2781 min)
PolyNet	NVIDIA GeForce RTX 3090	150 epochs, 50 batches/epoch, batch size 512	42.83 s (0.7139 min)
PolyNet-Volterra	NVIDIA GeForce RTX 3090	150 epochs, 50 batches/epoch, batch size 512	43.54 s (0.7256 min)

**Fig 21:**
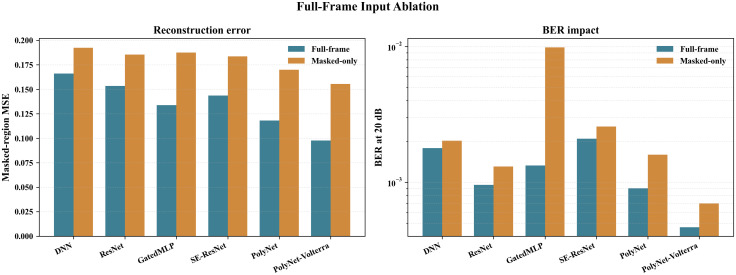
Full-frame input ablation comparing full-frame and masked-only neural reconstruction.

A full-frame input ablation was added by retraining the enabled neural models with masked-only inputs, where the unmasked samples were removed from the network input. Table 15 and Fig 21 show that masked-only input consistently increases masked-region MSE and BER for all tested neural models. This confirms that, without full-frame contextual information, the models lose important AFDM waveform features and the advantage of the proposed architecture is weakened.

**Table 15:** Full-frame input ablation at 20 dB.

Model	Input setting	BER mean ± SD	Masked MSE mean	MSE change
DNN	full-frame input	1.785e-03 ± 6.187e-04	0.1662	reference
DNN	masked-only input	2.023e-03 ± 1.456e-03	0.1924	+15.7%
ResNet	full-frame input	9.560e-04 ± 2.837e-04	0.1535	reference
ResNet	masked-only input	1.307e-03 ± 5.460e-04	0.1856	+20.9%
GatedMLP	full-frame input	1.330e-03 ± 6.021e-04	0.1339	reference
GatedMLP	masked-only input	9.848e-03 ± 1.527e-02	0.1876	+40.1%
SE-ResNet	full-frame input	2.096e-03 ± 1.490e-03	0.1437	reference
SE-ResNet	masked-only input	2.576e-03 ± 2.008e-03	0.1837	+27.8%
PolyNet	full-frame input	9.050e-04 ± 4.049e-04	0.1181	reference
PolyNet	masked-only input	1.595e-03 ± 6.846e-04	0.1700	+43.9%
PolyNet-Volterra	full-frame input	4.667e-04 ± 3.388e-04	0.0977	reference
PolyNet-Volterra	masked-only input	6.993e-04 ± 3.579e-04	0.1554	+59.1%

The receiver assumes conventional MMSE equalization with available channel information; synchronization errors and channel-estimation mismatch are important practical impairments that remain to be tested. For nonlinear RF impairments, Rapp/Saleh HPA models, memory-polynomial HPA behavior, spectral regrowth, and adjacent-channel leakage remain future validation tasks.

The present masking rule is based on time-domain amplitude and does not explicitly exploit DAFT-domain sparsity. Combining peak suppression with DAFT-domain sparse structure is a promising direction for adaptive masking design. The framework cannot be directly extended to OTFS without redesign. Only the general mask-and-reconstruct idea may be transferable; the input representation and physical feature basis would need to be rebuilt for the OTFS delay-Doppler transform structure.

A formal relation between masking sparsity and reconstruction-error bounds is not derived here. Such a bound would require assumptions on the AFDM signal distribution, channel model, masking pattern, and network approximation error. A learned adaptive masking policy may outperform the fixed threshold under changing SNR, channel, or modulation conditions. The fixed threshold is retained here because it provides a simple and reproducible setting for evaluating the reconstruction network.

## Conclusion

Addressing the severe PAPR problem encountered by AFDM systems in 6G high-mobility scenarios, this paper proposes a physics-aware mask-based PAPR reduction scheme that achieves both high energy efficiency and high reliability. To overcome the two major limitations of existing deep learning-based mask reconstruction models during signal recovery–namely, the lack of contextual information and unclear physical mechanisms–this paper first introduces a full-frame input strategy to thoroughly exploit the time-domain correlation of AFDM signals; secondly, it innovatively constructs the PolyNet-Volterra network based on the Volterra series theory. Through theoretical analysis and rigorous ablation studies, this paper breaks the conventional paradigm in the deep learning field of blindly stacking layers to enhance nonlinear fitting capabilities, establishing a minimalist network architecture that extracts exclusively the first-order linear terms and third-order power terms of the signal.

Simulation results demonstrate that the proposed masking mechanism effectively compresses signal peaks. Regarding signal reconstruction at the receiver, compared to traditional DNN and ResNet baseline models, the PolyNet-Volterra network accurately captures the core physical features of amplitude distortion. With an extremely low parameter count of approximately 160K, it not only effectively circumvents the overfitting risk introduced by higher-order features but also achieves a comprehensive outperformance in both reconstruction mean square error (MSE) and bit error rate (BER). This research provides a promising nonlinear inverse reconstruction approach for relieving the PAPR bottleneck in multi-carrier communication systems. The compact structure of PolyNet-Volterra also suggests possible value for future resource-constrained wireless receivers, such as vehicular, low Earth orbit satellite, and UAV-related scenarios. However, these applications still require hardware implementation, measured-channel evaluation, and over-the-air validation before practical deployment conclusions can be drawn.
